# Impaired degradation of YAP1 and IL6ST by chaperone-mediated autophagy promotes proliferation and migration of normal and hepatocellular carcinoma cells

**DOI:** 10.1080/15548627.2022.2063004

**Published:** 2022-04-18

**Authors:** Enrico Desideri, Serena Castelli, Coralie Dorard, Stefanie Toifl, Gian Luca Grazi, Maria Rosa Ciriolo, Manuela Baccarini

**Affiliations:** a Department of Biology, University of Rome “Tor Vergata”, Rome, Italy; b Department of Microbiology, Immunology and Genetics; Center of Molecular Biology, University of Vienna; Max Perutz Labs, Vienna, Austria; c Surgery Unit, Department of Clinical and Experimental Oncology, IRCCS - Regina Elena National Cancer InstituteHepato-Pancreato-Biliary, Rome, Italy; d IRCCS San Raffaele Pisana, Rome, Italy

**Keywords:** Chaperone-mediated autophagy, hepatocellular carcinoma, IL6ST, KFERQ motif, LAMP2A, migration, proliferation, protein degradation, YAP1

## Abstract

Impaired degradation of the transcriptional coactivator YAP1 and IL6ST (interleukin 6 cytokine family signal transducer), two proteins deregulated in liver cancer, has been shown to promote tumor growth. Here, we demonstrate that YAP1 and IL6ST are novel substrates of chaperone-mediated autophagy (CMA) in human hepatocellular carcinoma (HCC) and hepatocyte cell lines. Knockdown of the lysosomal CMA receptor LAMP2A increases protein levels of YAP1 and IL6ST, without changes in mRNA expression. Additionally, both proteins show KFERQ-dependent binding to the CMA chaperone HSPA8 and accumulate into isolated lysosomes after stimulation of CMA by prolonged starvation. We further show that LAMP2A downregulation promotes the proliferation and migration in HCC cells and a human hepatocyte cell line, and that it does so in a YAP1- and IL6ST-dependent manner. Finally, LAMP2A expression is downregulated, and YAP1 and IL6ST expression is upregulated, in human HCC biopsies. Taken together, our work reveals a novel mechanism that controls the turnover of two cancer-relevant proteins and suggests a tumor suppressor function of CMA in the liver, advocating for the exploitation of CMA activity for diagnostic and therapeutic purposes.

**Abbreviations:** ACTB: actin beta; ATG5: autophagy related 5; ATG7: autophagy related 7; CMA: chaperone-mediated autophagy; eMI: endosomal microautophagy; HCC: hepatocellular carcinoma; HSPA8: heat shock protein family A (Hsp70) member 8; IL6ST: interleukin 6 cytokine family signal transducer; JAK: Janus kinase; LAMP1: lysosomal associated membrane protein 1; LAMP2A: lysosomal associated membrane protein 2A; MAPK8: mitogen-activated protein kinase 8; P6: pyridine 6; SQSTM1: sequestosome 1; TUBA: tubulin alpha; VDAC1: voltage dependent anion channel 1; VP: verteporfin; YAP1: Yes1 associated transcriptional regulator.

## Introduction

Autophagy is a catabolic mechanism responsible for the degradation of cellular components via the lysosomal pathway and plays a pivotal role in maintaining protein homeostasis and protecting cells in stress conditions such as nutrient shortage or oxidative stress [1]. In mammalian cells, three primary forms of autophagy exist, each with its distinctive features: macroautophagy, in which double-membrane structures called phagophores engulf cell components and mature into autophagosomes, which then fuse with lysosomes to degrade their cargo; microautophagy, including endosomal microautophagy (eMI), in which proteins are imported into late endosomes or lysosomes; and chaperone-mediated autophagy (CMA), in which proteins translocate into the lysosomes through LAMP2A (lysosomal associated membrane protein 2A) [2]. Together with eMI, CMA is responsible for the degradation of proteins containing a recognition sequence named the KFERQ-like motif [3]. During CMA, the KFERQ motif is recognized by the chaperone HSPA8 (heat shock protein family A (Hsp70) member 8). The substrate-chaperone complex reaches the lysosome where it interacts with the CMA receptor LAMP2A and translocates into the lysosomal lumen [4]. Basal levels of CMA are detectable in unstimulated cells, but higher CMA activity is observed in response to stresses such as starvation, oxidative or genotoxic stress [5]. About 30% of cytosolic proteins contain KFERQ-like motifs and are therefore potential eMI or CMA substrates [6]. Aberrant degradation of these substrates can profoundly impact the cellular proteome, and reduced CMA activity has already been implicated in many pathological conditions, such as neurodegeneration, lysosomal storage disorders, and aging [5]. In cancer, the role of CMA is still controversial. LAMP2A has been observed elevated in many human cancer tissues, and CMA downregulation decreases the proliferation of lung and gastric cancer cells [7]. However, CMA also prevents fibroblast transformation by promoting the degradation of the MYC proto-oncogene [8] and many other cancer-related proteins, such as mutant TP53/p53 (tumor protein p53), LDHA (lactate dehydrogenase A), PKM (pyruvate kinase M1/2), and HK2 (hexokinase 2) are CMA substrates [9]. In the liver, CMA deficiency leads to metabolic dysregulation, hepatic glycogen depletion and hepatosteatosis [10], and restoration of CMA activity in aged mice reduces the accumulation of damaged proteins and improves liver function, highlighting hepatoprotective and possibly anti-tumor functions of CMA [11].

Hepatocellular carcinoma (HCC) is the primary liver malignancy and the third cause of cancer-related death worldwide. HCC incidence is high in Asia, Africa and Southern Europe, and it is one of the few cancer types whose mortality increased in the last decades [12]. Major risk factors for the development of HCC are persistent infections with hepatitis B and C viruses, alcohol abuse, obesity, exposure to toxins (e.g., aflatoxin B), and nonalcoholic fatty liver disease [12]. HCC is a highly heterogeneous cancer, in which aberrant activation of many oncogenic signaling pathways is observed with variable frequencies [13]. The transcriptional coactivator YAP1 (Yes1 associated transcriptional regulator) and IL6ST (interleukin 6 cytokine family signal transducer)-JAK (Janus kinase) signaling axis have a prominent role in tumor formation and growth, promoting cancer cell survival, proliferation, migration and metastasis [14,15]. They are coordinately upregulated at the post-translational level in mouse and human RAF1 knock-out HCC models, and responsible for the increased tumorigenesis induced by RAF1 ablation [16]. Impaired degradation of YAP1 and IL6ST by either the ubiquitin-proteasome system or autophagy was shown to favor the growth of different tumor types [17–21]. In this work, we provide evidence that CMA controls protein levels of YAP1 and IL6ST, which we validate as novel CMA substrates. Consistent with the pro-tumorigenic function of YAP1 and IL6ST in HCC, we also show that inhibition of CMA enhances proliferation and migration of a hepatocyte cell line and HCC cells in a YAP1- and IL6ST-dependent manner. Our work advances the current knowledge of mechanisms regulating the expression of cancer-related proteins, laying the groundwork for developing novel and specific strategies to be employed for therapeutic purposes.

## Results

### YAP1 and IL6ST are preferentially degraded by a lysosomal mechanism independent of macroautophagy in HCC cells

**Figure 1.**
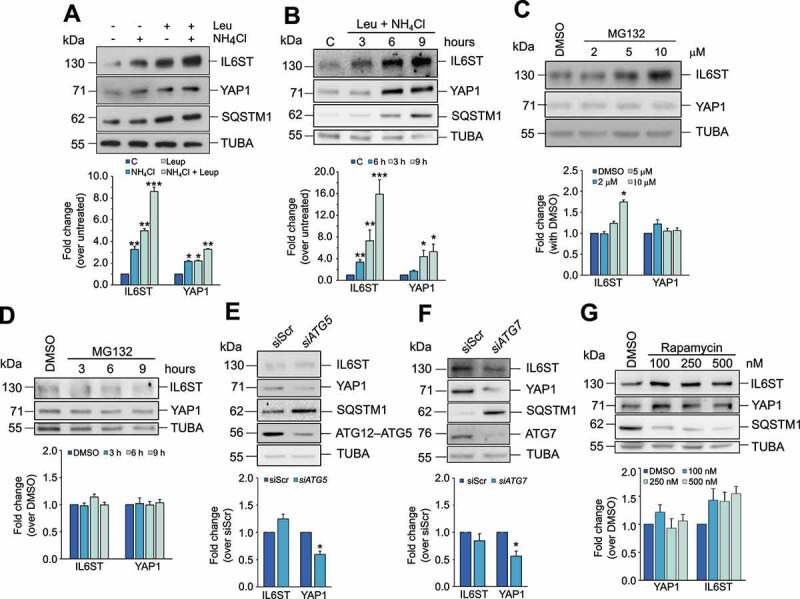
YAP1 and IL6ST are degraded via the lysosomal pathway. (**A,B**) Treatment of Hep3B cells for 6 h with the lysosomal inhibitor(s) NH_4_Cl (20 mM) and leupeptin (50 μM) (**A**) or with a combination of the two for the indicated time (**B**) leads to the accumulation of YAP1 and IL6ST in Hep3B cells. SQSTM1 is shown as a positive control. The bottom panel show the fold change of YAP1 and IL6ST over their expression in untreated cells, set as 1. (**C,D**) Treatment of Hep3B cells for 6 h with the indicated concentration of the proteasome inhibitor MG132 (**C**) or for the indicated time with 5 μM MG132 (**D**) has moderate to no effects on YAP1 and IL6ST levels. The bottom panel show the fold change of YAP1 and IL6ST over their expression in cells treated with DMSO, set as 1. (**E,F**) Silencing of either *ATG5* or *ATG7* does not cause the accumulation of YAP1 and IL6ST in Hep3B cells. SQSTM1 is shown as a positive control. The bottom panels show the fold change of YAP1 and IL6ST over their expression in cells transfected with siScr, set as 1. (**G**) Treatment of Hep3B cells for 24 h with the indicated concentrations of the macroautophagy-inducing drug rapamycin fails to reduce the levels of YAP1 and IL6ST in Hep3B cells. SQSTM1 is shown as a positive control. The bottom panel shows the fold change of YAP1 and IL6ST over their expression in cells treated with DMSO, set as 1. In all panels, the intensity of the bands of interest was normalized to TUBA prior to fold-change calculations. Data are plotted as the mean ± SEM of n = 3 biological replicates. *p < 0.05, **p < 0.01, ***p < 0.001 vs control.

To identify the mechanism through which YAP1 and IL6ST are preferentially degraded in HCC cells, we treated Hep3B cells either with lysosomal inhibitors (leupeptin, NH_4_Cl, or a combination of the two) or with the proteasome inhibitor MG132. Inhibition of lysosomal activity caused a much stronger increase in IL6ST and YAP1 expression (Figures 1A and 1B) than inhibition of the proteasome (Figures 1C, 1D; the efficacy of MG132 and absence of cell death are shown in **Figure S1A**). Similar results were obtained in the HCC cell line HepG2 and in the human hepatocyte cell line HuS (**Figures S1B** and **S1C)**. In the latter cell line, the effect of lysosomal inhibition on IL6ST was stronger than proteasome inhibition, while the inhibition of either mechanism had similar effects on YAP1, possibly reflecting a higher proteasomal turnover of YAP1 in normal than in HCC cells. In any case, these data demonstrate that lysosomes control the turnover of YAP1 and IL6ST in cells of liver origin. To verify whether YAP1 and IL6ST were degraded by macroautophagy, this process was either inhibited by siRNA against *ATG5* (autophagy related 5) or *ATG7* (autophagy related 7) or stimulated by rapamycin (Figure 1E and 1F). Inhibition of macroautophagy, clearly reflected in the accumulation of SQSTM1/p62 (sequestosome 1), did not increase YAP1 and IL6ST protein levels. Conversely, induction of macroautophagy, shown by SQSTM1 degradation, did not reduce YAP1 or IL6ST levels (Figure 1G). In contrast, inhibition of macroautophagy decreased YAP1 levels, and rapamycin treatment increased IL6ST levels. This phenomenon could be explained by a previously reported crosstalk between macroautophagy and other degradation pathways, such as CMA [22,23]. In Hep3B cells, modulation of macroautophagy had a negative impact on LAMP2A expression (**Figures S1D** and **S1E**), while LAMP2A silencing had a moderately positive effect on macroautophagy (**Figure S1F**). Be that as it may, the results are consistent with the idea that the lysosomal degradation of YAP1 and IL6ST is independent of macroautophagy.

### Modulation of CMA affects YAP1 and IL6ST levels

**Figure 2.**
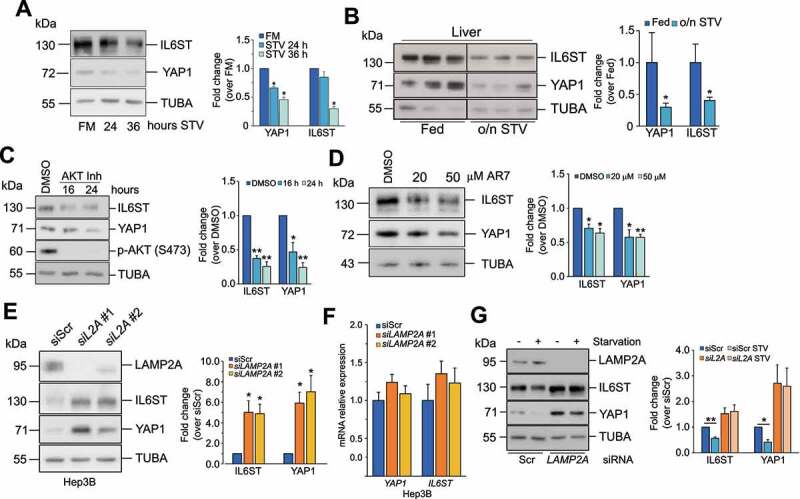
Modulation of CMA influences YAP1 and IL6ST protein levels. (**A**) Prolonged starvation reduces protein levels of YAP1 and IL6ST in Hep3B cells. The panel on the right shows the fold change of YAP1 and IL6ST over their expression in cells grown in FM, set as 1. (**B**) Overnight starvation reduces protein levels of YAP1 and IL6ST in mouse livers. The panel on the right shows the fold change of YAP1 and IL6ST over their expression in fed mice; lane 1 was set as 1. (**C**) Treatment of Hep3B cells with 10 μM of AKT inhibitor decreases YAP1 and IL6ST levels. The panel on the right shows the fold change of YAP1 and IL6ST over their expression in cells treated with DMSO, set as 1. (**D**) Treatment of Hep3B for 24 h with the indicated concentrations of the atypical RARA receptor antagonist AR7 decreases YAP1 and IL6ST levels. The panel on the right shows the fold change of YAP1 and IL6ST over their expression in cells treated with DMSO, set as 1. (**E**) Silencing of *LAMP2A* causes the accumulation of YAP1 and IL6ST in Hep3B cells. The panel on the right shows the fold change of YAP1 and IL6ST over their expression in cells transfected with siScr, set as 1. (**F**) Silencing of *LAMP2A* has no effect on the mRNA levels of *YAP1* and *IL6ST*. Data are plotted as the mean ± SEM of n = 3 biological replicates. *ACTB* was used as a housekeeping gene. (**G**) Silencing of *LAMP2A* prevents starvation-induced YAP1 and IL6ST degradation. Cells were cultured without serum for 24 h. The panel on the right shows the fold change of YAP1 and IL6ST over their expression in cells transfected with siScr, set as 1. The intensity of the bands of interest was normalized to TUBA (panels **A-E** and **G)**, prior to fold-change calculations. Data are plotted as the mean ± SEM of n = 3 biological replicates. *p < 0.05, **p < 0.01 vs control.

In addition to macroautophagy, lysosomes are the terminal station for other autophagic processes, such as CMA. Prolonged starvation, a condition that triggers CMA activation, led to a decrement of YAP1 and IL6ST levels in Hep3B cells and mouse livers (Figures 2A and B). Similarly, chemical stimulation of CMA by AKT1/2 (AKT serine/threonine kinase 1/2) inhibition [24] and the atypical RARA/RARα receptor (retinoic acid receptor alpha) antagonist, AR7 [25], caused a decrease of YAP1 and IL6ST (Figures 2C and 2D). Silencing of *LAMP2A*, the rate-limiting factor in CMA, lead to the accumulation of YAP1 and IL6ST proteins in Hep3B cells and in the human hepatocyte cell line HuS (Figures 2E and **S2A**) without significant changes in mRNA expression in either cell line (Figures 2F and **S2B**), and rescued starvation-induced decrease of both proteins (*siLAMP2A* #1 was used in this and all the following experiments) (Figure 2G). To clarify the contribution of CMA in the lysosomal degradation of YAP1 and IL6ST, we inhibited lysosomal activity in *LAMP2A*-silenced cells and measured the expression of the two proteins. The results showed that lysosomal inhibitors further increased IL6ST but not YAP1 levels (**Figure S2C**), indicating that CMA accounted for most of the lysosomal degradation of YAP1, while other lysosomal-dependent pathways appear to cooperate with CMA to regulate the turnover of IL6ST. Consistent with previous results, inhibition of the proteasome did not have any additive effect to CMA inhibition on either protein. Inhibition of lysosomal activity was also able to increase the expression of a YAP1^S127A^ mutant (**Figure S2D**), which is resistant to repression by the Hippo pathway [20,26]. *LAMP2A* knockdown had a similar effect (**Figure S2E**), while *ATG7* silencing did not promote the accumulation of either construct (**Figure S2F**), further confirming that lysosomal-dependent degradation of YAP1 relies on CMA. CMA downregulation has a milder effect on the YAP1^S127A^ mutant than on the WT YAP1. Serine 127 phosphorylation is important for the retention of YAP1 into the cytoplasm [20,26]. Therefore, this result may reflect a reduced accessibility of the YAP1^S127A^ mutant to the CMA machinery. Although CMA has already been shown to degrade nuclear proteins (e.g. CHEK1/Chk1) [27], it may target them less efficiently than cytosolic substrates. Importantly, *LAMP2A* silencing in Hep3B and HuS cells promoted YAP1 nuclear translocation and increased the expression of *CCN2/CTGF* (cellular communication network factor 2) (YAP1 target gene) and *BIRC5* (baculoviral IAP repeat containing 5) (common YAP1-IL6ST pathway target) (**Figures S2G-S2J**). Together, these results demonstrate that CMA modulation impacts the levels of YAP1 and IL6ST as well as their output.

### YAP1 and IL6ST are bona fide substrates of CMA

**Figure 3.**
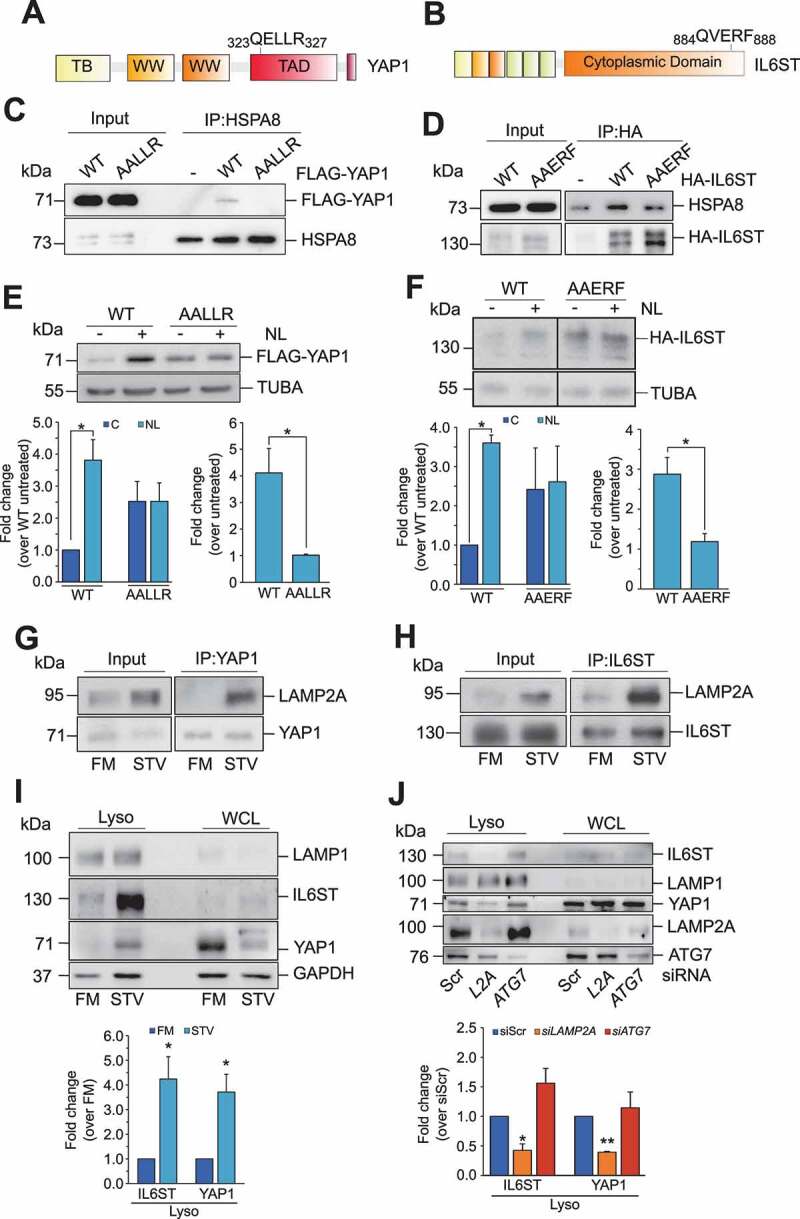
YAP1 and IL6ST are bona fide CMA substrates. (**A,B**) Graphical representation of the position of the putative KFERQ motif within the human YAP1 (**A**) and IL6ST (**B**) sequences. (**C**) FLAG-YAP1 WT, but not FLAG YAP1 AALLR, is detectable in HSPA8 immunoprecipitates. (**D**) More HSPA8 is detectable in HA-IL6ST WT than in IL6ST AAERF immunoprecipitates. (**E,F**) Mutation of the KFERQ motif increases the expression of YAP1 (**E**) and (**F**) IL6ST and renders them insensitive to treatment with lysosomal inhibitors (NL; 20 mM NH_4_Cl and 50 μM leupeptin, 6 h). The left bottom panel shows the fold change of YAP1 (**E**) and IL6ST (**F**) over the expression of the untreated WT, set as 1. The right bottom panel shows the fold change of YAP1 (**E**) and IL6ST (**F**) over the expression of the same construct in the untreated cells, set as 1. (**G,H**) Starvation induces complex formation between YAP1 (**G**) or IL6ST (**H**) and LAMP2A. (**I**) Starvation promotes the accumulation of YAP1 and IL6ST, and the canonical CMA substrate GAPDH in lysosomes isolated from Hep3B cells. The bottom panel shows the fold change of YAP1 and IL6ST over their expression in cells grown in FM. (**J**) Silencing of *LAMP2A*, but not *ATG7*, reduces starvation-induced lysosomal localization of YAP1 and IL6ST. Cells were cultured without serum for 24 h. The bottom panel shows the fold change of YAP1 and IL6ST over their expression in cells transfected with siScr. The intensity of the bands of interest was normalized to TUBA (panels **E** and **F**) or LAMP1 (panels **I** and **J**) prior to fold-change calculations. Data are plotted as the mean ± SEM of n = 3 biological replicates. *p < 0.05, **p < 0.01, ***p < 0.001 vs control.

Next, we investigated whether YAP1 and IL6ST were substrates of CMA. To be validated as a CMA substrate, a protein must satisfy established criteria, described elsewhere [28]. We have already shown that protein levels of YAP1 and IL6ST were increased by *LAMP2A* silencing and decreased by conditions activating CMA. In addition, all CMA substrates must contain a KFERQ-motif, recognized by HSPA8 and necessary for the lysosomal translocation of the substrates. Sequence analysis revealed that both YAP1 and IL6ST featured a putative KFERQ motif (Figures 3A and B). Mutation of the KFERQ motif reduced the interaction of YAP1 and IL6ST with HSPA8 (Figures 3C and D). The basal expression levels of the KFERQ mutants were higher than those of the corresponding WT, although not significantly so (compare lanes 1 with lane 3, Figure 3E and 3F; see also left bottom panels). More importantly, mutation of the KFERQ motifs dramatically reduced the accumulation of YAP1 and IL6ST in response to treatment with inhibitors of lysosomal activity (NL: NH_4_Cl and leupeptin) (Figures 3E and F; compare the fold change in the expression of the WT and KFERQ mutant in cells untreated or treated with NL, right bottom panel). These data imply that CMA is the main lysosomal degradation mechanism for these two proteins. In line with this, stimulation of CMA by prolonged starvation also promoted the interaction of both proteins with LAMP2A (Figures 3G and H) and their accumulation into the lysosomes (Figure 3I; purity of lysosomal fraction is shown in **Figure S3**). Knockdown of *LAMP2A*, but not of *ATG7*, reduced starvation-induced lysosomal accumulation of YAP1 and IL6ST (Figure 3J), further confirming that macroautophagy was not involved in this process. Overall, these data indicate that YAP1 and IL6ST are novel substrates of CMA.

### LAMP2A silencing promotes proliferation and migration of normal and HCC cells in a YAP1 and IL6ST-dependent manner

**Figure 4.**
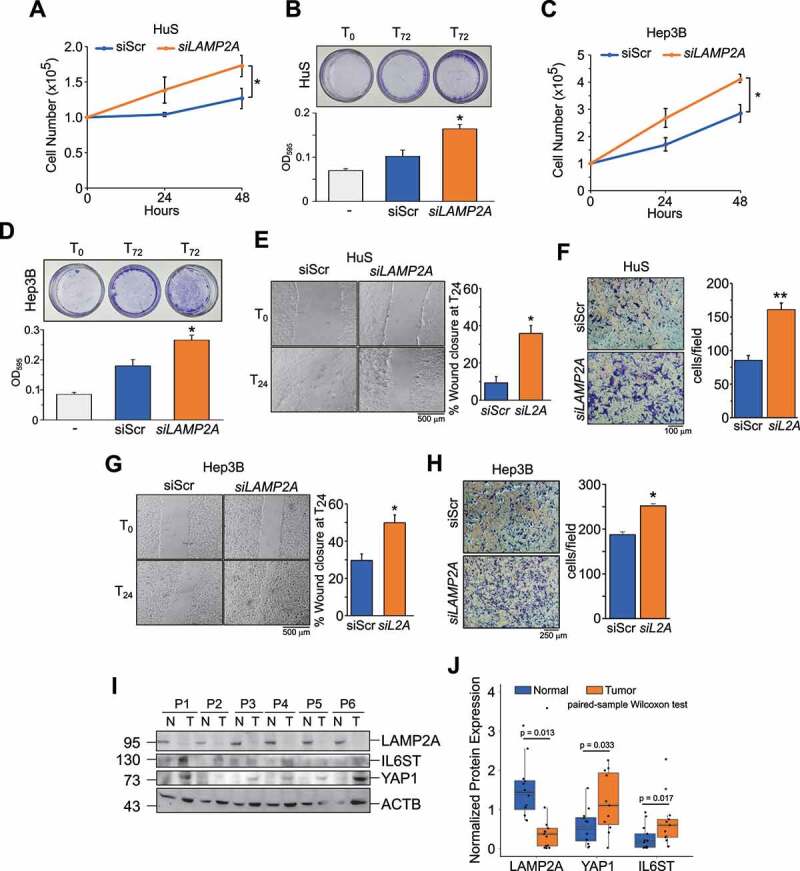
Inhibition of CMA promotes cell proliferation and migration. (**A,B**) Inhibition of CMA increases proliferation of HuS cells, measured by live cell counting (**A**) and crystal violet staining (**B**). (**C,D**) Inhibition of CMA increases proliferation of Hep3B cells, measured by cell counting (**C**) and crystal violet staining (**D**). (**E,F**) Inhibition of CMA increases migration of HuS cells, measured by the wound healing (**E**) and transwell (**F**) assays. (**G,H**) Inhibition of CMA increases migration of Hep3B cells, measured by the wound healing (**G**) and transwell (**H**) assays. Data are plotted as the ± SEM of n = 3 biological replicates. *p < 0.05, **p < 0.01 vs siScr. (**I**) LAMP2A, YAP1 and IL6ST protein expression in human HCC samples (T) compared to matched adjacent normal tissues (N). (**J**) Box and whiskers plot of densitometry analysis of LAMP2A, YAP1 and IL6ST protein expression normalized to ACTB in n = 12 human HCC samples and matched adjacent normal tissues. Data are compared using the paired samples Wilcoxon test for not normally distributed data.

Both IL6ST and YAP1 have been shown to promote tumorigenic features of tumor cells, such as proliferation and migration [15,29]. If, as indicated above, these proteins are CMA substrates, inhibition of CMA might also affect these processes. To test this hypothesis, we employed one human hepatocyte cell line, HuS, and two HCC cell lines, Hep3B and HepG2, in which CMA was downregulated by *LAMP2A* knockdown (**Figures S4A-S4C**). Live cell counting (Figure 4A) and crystal violet staining (Figure 4B) showed that LAMP2A downregulation significantly increased cell proliferation in HuS cells. Analogous results have been obtained in Hep3B (Figures 4C and D) and HepG2 cells (**Figures S4D**). Next, we analyzed the effect of LAMP2A downregulation on cell migration using both wound healing and transwell assays. As shown in Figures 4E and 4F, *LAMP2A*-silenced HuS cells showed increased migration compared to control cells. Similar results were observed in Hep3B (Figures 4G and H) and HepG2 cells (**Figures S4E** and **S4F**). Thus, reducing the levels of LAMP2A promoted proliferation and migration, two hallmark features of tumor cells, suggesting an anti-tumorigenic function of LAMP2A and CMA. In support of this hypothesis, immunoblot analysis of biopsies of HCC patients clearly showed lower expression of *LAMP2A* in tumor tissues compared to adjacent non-tumor tissues (Figures 4I, 4J and **Figure S4G**). In the same samples, the expression of *YAP1* and *IL6ST* was instead increased, consistent with the results obtained in cultured liver cell lines.

**Figure 5.**
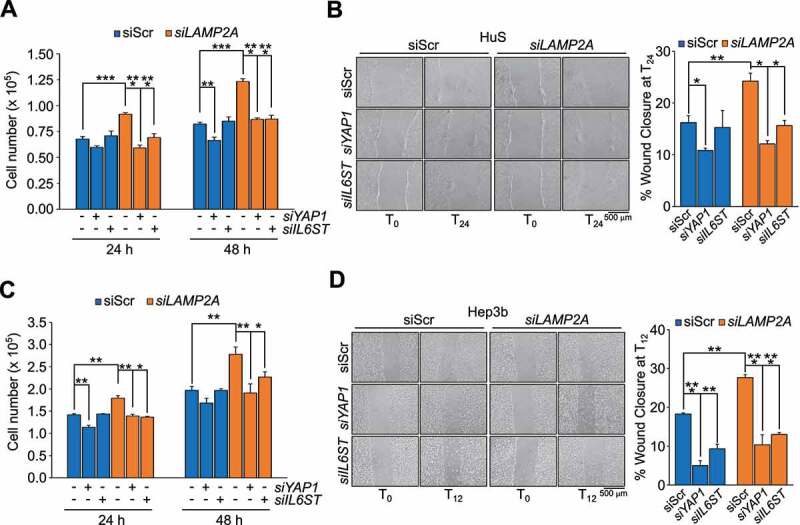
YAP1 and IL6ST contribute to the growth advantage and increased motility induced by CMA downregulation. (**A,C**) Silencing of either *YAP1* or *IL6ST* restrains the increased proliferation of *LAMP2A* knockdown HuS (**A**) and Hep3B (**C**) cells. (**B,D**) Silencing of either *YAP1* or *IL6ST* restrains the increased migration of *LAMP2A* knockdown HuS (**B**) and Hep3B (**D**) cells, measured by the wound healing assay. Data are plotted as the ± SEM of n = 3 biological replicates. *p < 0.05, **p < 0.01, ***p < 0.001 vs siScr.

To address whether YAP1 and IL6ST contributed to the proliferative phenotype of CMA impaired cells we tested whether silencing these genes would rescue the phenotypes of *LAMP2A* knockdown cells (**Figures S5A** and **S5B**). In HuS and Hep3B cells, silencing of *YAP1* or *IL6ST* efficiently reverted the increased proliferation of *LAMP2A*-knockdown cells (Figures 5A and 5C). Similar results were obtained using verteporfin (VP) and the pan JAK inhibitor pyridone 6 (P6), inhibitors of YAP1 and IL6ST-JAK signaling, respectively (**Figures S5C** and **S5E)**. Analysis of migration by the wound healing assay also showed that silencing of either *YAP1* or *IL6ST* reverted the increased migration of *LAMP2A* knockdown HuS and Hep3B cells (Figures 5B and 5D). Again, similar results were obtained using VP and P6 (**Figures S5D** and **S5F)**. Collectively, these data demonstrated that YAP1 and IL6ST contributed to the positive effect of CMA downregulation on cell proliferation and migration.

## Discussion

Many cellular functions, such as cell cycle progression and signal transduction, are characterized by a finely regulated fluctuation of protein levels, determined by a balance between gene transcription, protein synthesis, and protein degradation. In eukaryotic cells, the activity of the ubiquitin-proteasome system and autophagy determine protein turnover, removing defective or misfolded proteins and providing amino acids for de novo protein synthesis, thus maintaining homeostasis [30]. Loss of protein homeostasis can lead to abnormal protein expression, altering cell functions, and it is linked to a wide range of diseases, including cancer [31–33].

In this study, we demonstrate that CMA controls the levels of YAP1 and IL6ST, expanding the range of cancer-relevant proteins whose turnover is controlled by CMA [9]. Silencing of the CMA receptor *LAMP2A* increased the levels of both proteins without changes in mRNA expression, and further analysis demonstrated that YAP1 and IL6ST were direct CMA substrates, revealing a novel mechanism that controls the expression of two cancer-relevant proteins. In fact, YAP1 and IL6ST signaling pathways have a role in promoting liver tumors [29,34], and the impaired degradation of the two proteins by either the proteasome or macroautophagy has already been shown to promote tumor growth [17–19,21,35]. In line with the identification of YAP1 and IL6ST as CMA substrates in liver cells, we could show that CMA downregulation promoted proliferation and migration in a hepatocyte cell line and HCC cell lines. Genetic or pharmacological inhibition of YAP1 and IL6ST restrained the growth advantage of *LAMP2A*-knockdown cells, demonstrating that the impaired degradation of the two proteins by CMA has a significant role in the phenotype imposed by *LAMP2A* silencing. Interestingly, inhibition of YAP1 and IL6ST had a cell line-specific effect on the proliferation of WT cells, with the HuS cell showing little sensitivity. When *LAMP2A* was silenced, however, both cell lines tested responded in a similar manner to inhibition of either pathway. In light of this, LAMP2A expression may represent a predictor of cell sensitivity to specific inhibitors and eventually find application in biomarker-driven anticancer therapy. This aspect is particularly important in HCC, in which the high heterogeneity and the high number of oncogenic signaling pathways potentially involved in tumor growth make the identification of feasible therapeutic strategies challenging [36].

The demonstration that CMA modulation controls the turnover of two cancer proteins and, in turn, influences cell proliferation and migration argues for a tumor suppressor role of CMA in the liver. The role of CMA in cancer is still unclear, with numerous lines of evidence arguing for a pro- or an anti-tumorigenic function of this mechanism, depending on the cell and tumor type [7,37–39]. In support of the hypothesis that CMA acts as a tumor suppressor in the liver, LAMP2A expression was reduced in HCC biopsies compared to their matched adjacent normal tissues, and an anticorrelation with YAP1 and IL6ST could be observed. A tumor suppressor function of CMA in the liver can also be envisaged from the work of the Cuervo´s group, showing that deficient CMA leads to liver metabolic dysregulation and hepatosteatosis [10], emerging as risk factors for HCC in developed countries [40]; and that restoration of CMA activity in aged mice reduces the accumulation of damaged proteins and improves liver function [11]. Stimulation of CMA could be deployed in cancer prevention to prevent aberrant protein accumulation and maintain protein homeostasis, for instance in people with a higher than average risk of developing liver cancer. In this context, activators of CMA have already been characterized in vitro and in vivo [25,41]. In particular, the mitochondrial-derived peptide humanin and its analogs were shown to protect cells from a multitude of stress conditions and exert cardioprotective and neuroprotective functions in vitro and in vivo [41,42]. CMA mediates the cytoprotective effects of humanin in vitro [41], but whether and to which extent this also occurs in vivo is still to be clarified. In conclusion, our work expands the list of cancer-related CMA substrates, advocating for the exploitation of CMA activity for diagnostic and therapeutic purposes.

## Materials and methods

### Cell culture

Hep3B and HepG2 cell lines were purchased from the Leibniz-Institut DSMZ, Braunschweig (ACC-93, ACC-180) and grown in DMEM (Euroclone, ECM0749L) and RPM1640 (Euroclone, ECB9006L), respectively, supplemented with 10% FBS (Euroclone, ECS0180L) and 1% penicillin-streptomycin. HuS cells were a gift of Dr. Vinicio Carloni, University of Florence and were grown in DMEM containing 4.5 g/L of glucose (Euroclone, ECB7501L) and supplemented with 10% FBS (Euroclone, ECS0180L), 5 ng/mL EGF (Sigma-Aldrich, E9644), 420 ng/mL insulin (Sigma-Aldrich, I9278), 20 ng/mL selenium (Sigma-Aldrich, S9133), 1% DMSO (Amresco, 0231) and 1% penicillin-streptomycin (Euroclone, ECB3001D). Cells have been authenticated by STR PCR by the supplier and routinely tested for Mycoplasma contamination using Myco Alert (Lonza, LT07-318).

### Treatments

Lysosomal inhibitors NH_4_Cl (Sigma-Aldrich, A9434) and leupeptin (Sigma-Aldrich, SAE0153) were used at the final concentration of 20 mM and 50 μM, respectively. Proteasome inhibitor MG132 was purchased from Sigma-Aldrich (474791) and used at the concentrations indicated in the text. Rapamycin was purchased from Enzo Lifesciences (BML-A275-0005) and used at the concentrations indicated in the text. AKT inhibitor VIII was purchased from Sigma-Aldrich (124017) and used at the final concentration of 10 μM. The atypical RARA receptor antagonist AR7 was purchased from Sigma-Aldrich (SML0921) and used at the indicated concentrations. YAP1 inhibitor verteporfin (VP) was purchased from Sigma-Aldrich (SML0534) and used at the final concentration of 5 μM. Pan JAK inhibitor pyridone 6 (P6) was purchased by Sigma-Aldrich (420097) and used at the final concentration of 1 μM.

### Patient samples

Human HCC biopsies were provided by Prof. Grazi from the Hepato-Pancreato-Biliary Surgery Unit, Department of Clinical and Experimental Oncology, Regina Elena National Cancer Institute, Rome, Italy. Analysis was performed after approval from the Regina Elena Cancer Institute ethics committee.

### Animal experiments

Male mice on an Sv/129 background were food-deprived overnight, euthanized with CO_2,_ and livers collected. Animal experiments were authorized by the Austrian Ministry of Science, Research and Economy.

### Transfection

SiRNAs and esiRNA were transfected using Lipofectamine RNAiMax (Life Technologies, 3778075). The following siRNAs and esiRNA (all from Sigma-Aldrich) were used: *LAMP2A#1* (5´-UUACCUCUCAGUUGUUGAA-3´); *LAMP2A#2* (5´-CCAUCAUGCUGGAUAUGA-3´); *ATG7* (5´-ATGGAGAGCTCCTCAGCAGGC-3´); *YAP1* (5´-TTCTTTATCTAGCTTGGTGGC-3´); *IL6ST* (5´-AATGTGAAATATCTGGACTGG-3´); siRNA Universal Negative Control (SIC001) was used as negative control; *ATG5* (EHU085781); *RLuc* (EHURLUC) was used as esiRNA negative control. Unless otherwise stated, all siRNAs were used at the final concentration of 25 nM and cells assayed 48 h after transfection. Plasmids were transfected using Lipofectamine 3000 following the manufacturer’s instructions (Life Technologies, L3000001). pCMV3-N-HA-GP130 (IL6ST; HG10974-NY) was purchased from Sino biological Inc, while the p2xFlag CMV2-YAP2 and the p2xFlag CMV2-YAP2-S127A plasmids were a gift from Marius Sudol (Addgene, 19,045; http://n2t.net/addgene:19045; RRID:Addgene_19045; Addgene, 19050; http://n2t.net/addgene:19050; RRID:Addgene_19050) [43].

### Immunoblotting and co-immunoprecipitation

Immunoblotting was carried out as previously described [16]. The following antibodies were used at a dilution of 1:1000 unless otherwise stated: ATG5 (8540), ATG7 (8558), SQSTM1 (8025), YAP1 (4912), p-AKT (Ser473; 9271), PARP1 (9542) and FLAG (2368), all from Cell Signaling Technology; ACTB (1:3000; sc-8432), IL6ST (1:500; sc-656), LAMP1 (1:2000; sc-20011), LMNB1 (sc-377000), ACTN (sc-17829), MAPK8 (sc-137018), and VDAC1 (sc-390996), all from Santa Cruz Biotechnology. HSPA8 (ADI-SPA-815-F) from Enzo Life Science; LAMP2A (1:3000; ab18528) and HA-Tag (ab9110) from Abcam; Ubiquitin (MAB1510) and TUBA (1:5000; T9026) from Sigma-Aldrich. In co-immunoprecipitation experiments, cells were lysed in lysis buffer (140 mM KCl [Sigma-Aldrich, 104936], 3 mM MgCl_2_ [Sigma-Aldrich, 442615], 1% NP-40 [Sigma-Aldrich, 74385], 20 mM HEPES [Sigma-Aldrich, H23830], pH 7.4, 1 mM EDTA [Sigma-Aldrich, 03609], 1.5 mM EGTA [Sigma-Aldrich, E4378]) supplemented with 10 mM NaF (Sigma-Aldrich, S7920), 1 mM Na_3_VO_4_ (Sigma-Aldrich, 567540) and protease inhibitor cocktail (Roche, P8849) and centrifuged at 20,000 × g for 15 min. Five hundred μg of protein lysate were incubated overnight at 4°C with 2 μg of primary antibody, followed by 4 h of incubation with 50 μl of Rec-Protein G – Sepharose® 4B beads (Thermo Scientific, 101241). An isotype control antibody (Cell Signaling Technology, 3900) was used as a negative control. Beads were washed three times in lysis buffer, incubated for 6 min at 95°C in Laemmli sample buffer (Bio-Rad Laboratories, 1610737) and subjected to SDS-PAGE. Immunoblots (representative of at least 2 experiments) were acquired using ChemiDoc Imaging Systems and quantified using the Image Lab software (Bio-Rad Laboratories, Hercules, USA).

### Site-directed mutagenesis

Mutation of the KFERQ sequence of YAP1 and IL6ST was performed using the QuikChange site-directed mutagenesis kit (Stratagene, 200522), using the following primers:

*YAP1*: 5’-CTGCGGCTGAAACAGGCCGCCCTGCTTCGGCAGGAGTTAG-3’;

*IL6ST*: 5’-ACTGTTTCAAATCTTTCTGCTGCTCCCTCAGTACCTGGACCAAAAGC-3’. The presence of the desired mutations and the absence of additional mutations within the coding sequence was confirmed by DNA sequencing.

### Real-time qPCR

Cells were homogenized in TRI Reagent (Sigma-Aldrich, T9424) and RNA was extracted according to the manufacturer’s instructions. Total RNA was resuspended in RNase-free water (Sigma-Aldrich, W4502) and 1 µg of total RNA was used to generate cDNA using the PrimeScript RT-PCR Kit (Takara Bio, RR014A). Real-time PCR was performed using the PowerUp SYBR Green Master Mix (Thermo Fisher Scientific, A25742) on a QuantStudio 3 Real-Time PCR system (Thermo Fisher Scientific, Waltham, USA). The following primer pairs (purchased from Sigma-Aldrich) were used: *YAP1* (Forward 5´-CCCGACAGGCCAGTACTGAT-3´, Reverse 5´- CAGAGAAGCTGGAGAGGAATGAG-3´); *IL6ST* (Forward 5´- GACCATCTAAAGCACCAAGTTTCT-3´, Reverse 5´- AAAGGAGGCAATGTCTTCCACA-3´); *ACTB* (Forward 5´-GGCCGAGGACTTTGATTGCA-3´, Reverse 5´- GGGACTTCCTGTAACAACGCA-3´); *BIRC5* (Forward 5’-CTTTCTCAAGGACCACCGCA-3’, Reverse 5’-CTCGGCCATCCGCTCC-3’); *CCN2* (Forward 5’-CCTTCCCGAGGAGGGTCAA-3’, Reverse 5’-CAGTCGGTAAGCCGCGAG-3’). All reactions were run as triplicates. Data were analyzed by the Design and Analysis Application (Thermo Fisher Scientific) using the second derivative maximum method. The fold changes in mRNA levels were relative to the control after normalization to *ACTB*.

### Lysosome isolation

Lysosomes were isolated from a light mitochondrial fraction using a discontinuous Nycodenz (ProteoGenix, 1002424) gradient. To activate CMA, cells were incubated in serum-free medium for 24 h. Cells were resuspended in a homogenization medium (HM; 0.25 M sucrose [Sigma-Aldrich, S0389], 1 mM EDTA [Sigma-Aldrich, 03609], 10 mM HEPES [Sigma-Aldrich, H23830], pH 7.4) containing 10 mM NaF (Sigma-Aldrich, S7920), 1 mM Na_3_VO_4_ (Sigma-Aldrich, 567540) and protease inhibitor cocktail (Roche, P8849) and mechanically disrupted using a Dounce homogenizer (35 strokes; VWR, WHEA357538). After centrifugation at 750 x g for 10 min at 4°C, the supernatant was collected and centrifuged at 20,000 x g for 10 min at 4°C to pellet the light mitochondrial fraction, which contains lysosomes. The pellet was resuspended in HM and 2 volumes of 45% Nycodenz (ProteoGenix, 1002424) were added (30% Nycodenz final concentration). The sample was loaded on the bottom of a discontinuous Nycodenz gradient (30%, 26%, 24%, and 19%) and centrifuged at 100,000 x g for 2 h at 4°C using a swinging bucket rotor (SW41Ti; Beckman Coulter, 331362). Lysosomes were recovered from the 19%-24% interface, diluted in 3 volumes of PBS (137 mM NaCl [Sigma-Aldrich, S9625], 2.7 mM KCl [Sigma-Aldrich, 104936], 10 mM Na_2_HPO_4_ [Sigma-Aldrich, S0876], and 1.8 mM KH_2_PO_4_ [Sigma-Aldrich, P0662], pH 7.4) to reduce the Nycodenz concentration, and centrifuged at 25,000 x g for 30 min at 4°C. The pellet was used for SDS-PAGE. To enrich for lysosomal substrates, cells were incubated for 24 h with lysosomal inhibitors leupeptin (Sigma-Aldrich, SAE0153) and NH_4_Cl (Sigma-Aldrich, A9434).

### Nuclei isolation

Cells were lysed in a nucleus extraction buffer (NB; 0.25 M sucrose [Sigma-Aldrich, S0389], 10 mM Tris [Sigma-Aldrich, 93352], pH 8, 10 mM MgCl_2_ [Sigma-Aldrich, 442,615], 1 mM EDTA [Sigma-Aldrich, 03609], 1% Triton X-100 [Sigma-Aldrich, T9284], 0.5 mM DTT [Bio-Rad Laboratories, 1,610,611) containing 10 mM NaF (Sigma-Aldrich, S7920), 1 mM Na_3_VO_4_ (Sigma-Aldrich, 567540) and protease inhibitor cocktail (Roche, P8849). After centrifugation at 600 x g for 10 min at 4°C, supernatants were transferred to new tubes and pellets containing nuclei were resuspended with NB and centrifuged at 600 x g for 10 min at 4°C. Pellets were then lysed in RIPA buffer (50 mM Tris [Sigma-Aldrich, 93,352], pH 8.0, 150 mM NaCl [Sigma-Aldrich, S9625], 1% NP40 [Sigma-Aldrich, 74,385], 0.1% SDS [Sigma-Aldrich, 8170341000], 0.5% sodium deoxycholate [Sigma-Aldrich, D6750]) supplemented with 10 mM NaF (Sigma-Aldrich, S7920), 1 mM Na_3_VO_4_ (Sigma-Aldrich, 567540) and protease inhibitor cocktail (Roche, P8849) and processed for SDS-PAGE.

### Live cell counting

Cells were transfected as required and re-seeded after 12 h. After 12 h (T_0h_), 36 h (T_24h_) and 60 h (T_48h_), cells were collected and live cells counted after staining with 0.08% Trypan Blue (Sigma-Aldrich, T8154). Where required, inhibitors were added at T_24_ and cells counted after an additional 24 h.

### Crystal violet staining

Cells transfected with either a siScr or si*LAMP2A* were plated in 35-mm dishes and let grow for 72 h. At the end of the experimental time, cells were fixed for 15 min with ice-cold methanol, washed with PBS (137 mM NaCl [Sigma-Aldrich, S9625], 2.7 mM KCl [Sigma-Aldrich, 104936], 10 mM Na_2_HPO_4_ [Sigma-Aldrich, S0876], and 1.8 mM KH_2_PO_4_ [Sigma-Aldrich, P0662], pH 7.4) and stained with 0.5% crystal violet (Sigma Aldrich, 61135) for 30 min at room temperature. After three washes with PBS, dishes were air-dried and images acquired using a digital camera. Finally, crystal violet was eluted with 10% acetic acid (Sigma-Aldrich, A6283) and quantification was performed by measuring the absorbance at 595 nm using a microplate reader.

### Wound healing assay

Cells were transfected as required and let grow until confluent. Then, cells were serum-starved overnight to prevent proliferation, medium replaced with one containing 1% FBS (Euroclone, ECS0180L) and a wound created using a pipette tip. Images were taken using an inverted microscope connected with a digital camera at the beginning (T_0_) and end of the experimental time (T_12-24h_). Quantification of gap closure was performed using the ImageJ software. In selected experiments inhibitors were added 1 h before T_0_.

### Transwell migration assay

Cells were transfected with either a siScr or *siLAMP2A* and let grow for 48 h, after which time they were trypsinized, counted and resuspended in serum-free medium. SiScr and si*LAMP2A* cells (1 x 10^5^) were transferred to transwell inserts with 8-μm pore size (VWR, 734–1574). The outer chambers were filled with complete medium. After 16 h, cells were fixed with 10% formalin solution (Sigma-Aldrich, HT5011) and stained with 0.5% crystal violet (Sigma Aldrich, 61135) for 30 min at room temperature. Non-migrated cells were removed with a cotton swab and images were taken using an inverted microscope connected with a digital camera. Migrated cells were counted using the ImageJ software.

### Statistical analysis

Values are expressed as means ± SEM of at least 3 independent experiments; unless otherwise indicated, p values were calculated using the two-tailed Student’s t-test. A p-value < 0.05 is considered statistically significant and a p-value < 0.01 is considered highly statistically significant.

## Supplementary Material

Supplemental Material
