# Supplementary material for: Impaired degradation of YAP1 and IL6ST by chaperone-mediated autophagy promotes proliferation and migration of normal and hepatocellular carcinoma cells
Source: Autophagy. 2022 Apr 18;19(1):152–62. doi: 10.1080/15548627.2022.2063004 (PMC9809932; doi:10.1080/15548627.2022.2063004)
Supplement: Supplemental Material [file KAUP_A_2063004_SM1123.docx]

**
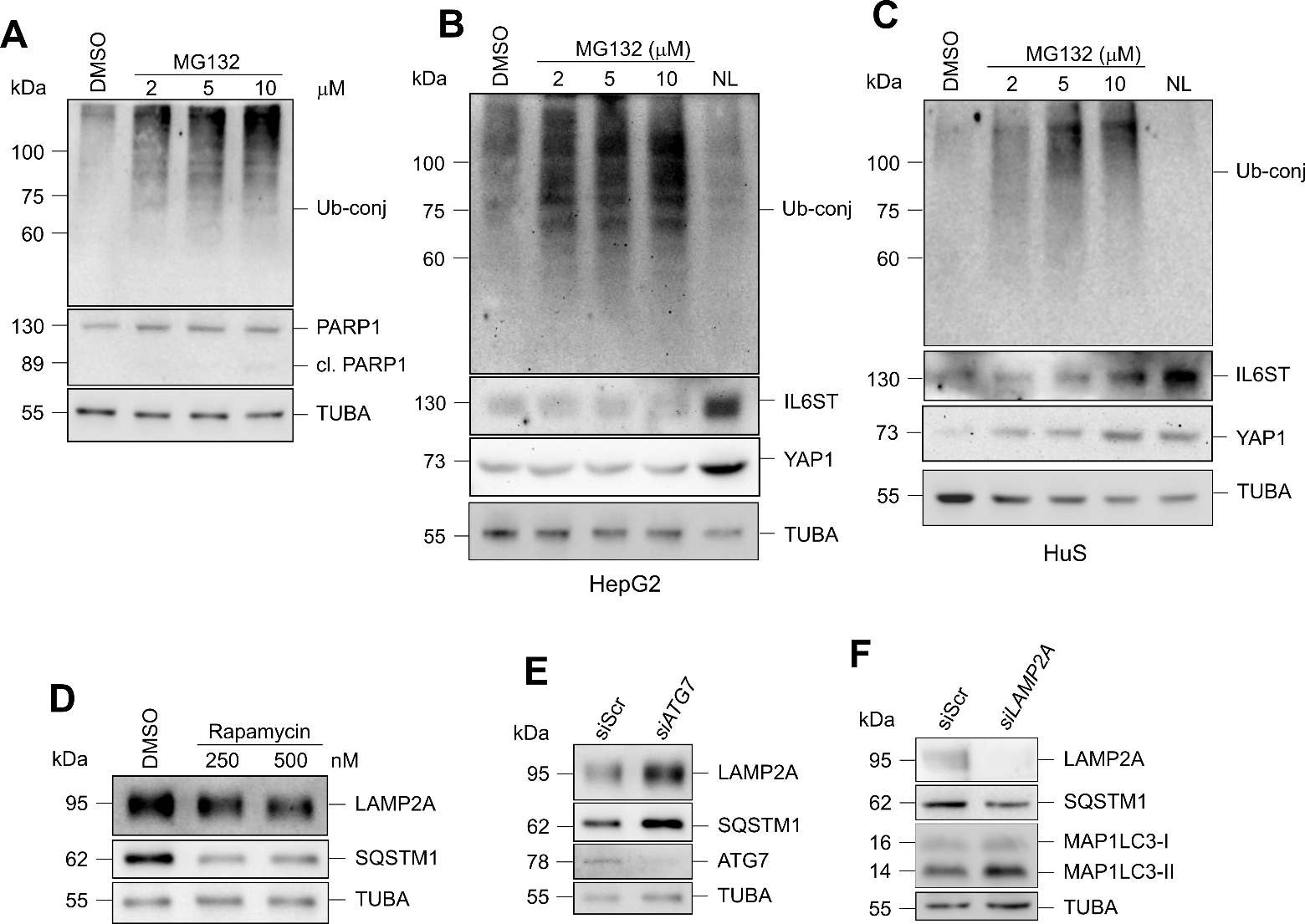
**

**Figure S1.** YAP1 and IL6ST are degraded via the lysosomal pathway. (**A**) Western blot analysis of ubiquitin-conjugated proteins (Ub-conj) and apoptosis marker PARP1 in Hep3B cells treated for 6 h with the indicated concentrations of MG132. (**B-C**) Western blot analysis of ubiquitin-conjugated proteins, YAP1 and IL6ST in HepG2 (**B**) and HuS (**C**) cells treated for 6 h either with the indicated concentrations of MG132 or with a combination of 50 μM leupeptin and 20 mM NH_4_Cl. (**D**) Treatment of Hep3B cells for 24 h with the indicated concentrations of the macroautophagy-inducing drug rapamycin reduces the levels of LAMP2A. SQSTM1 reduction is shown as a positive control (**E**) Silencing of *ATG7* increases LAMP2A in Hep3B cells. SQSTM1 accumulation is shown as a positive control. (**F**) Silencing of *LAMP2A* causes a moderate decrease of SQSTM1 and an increase of MAP1LC3-II, compatible with an increased autophagic flux. Related to Figure 1.

**
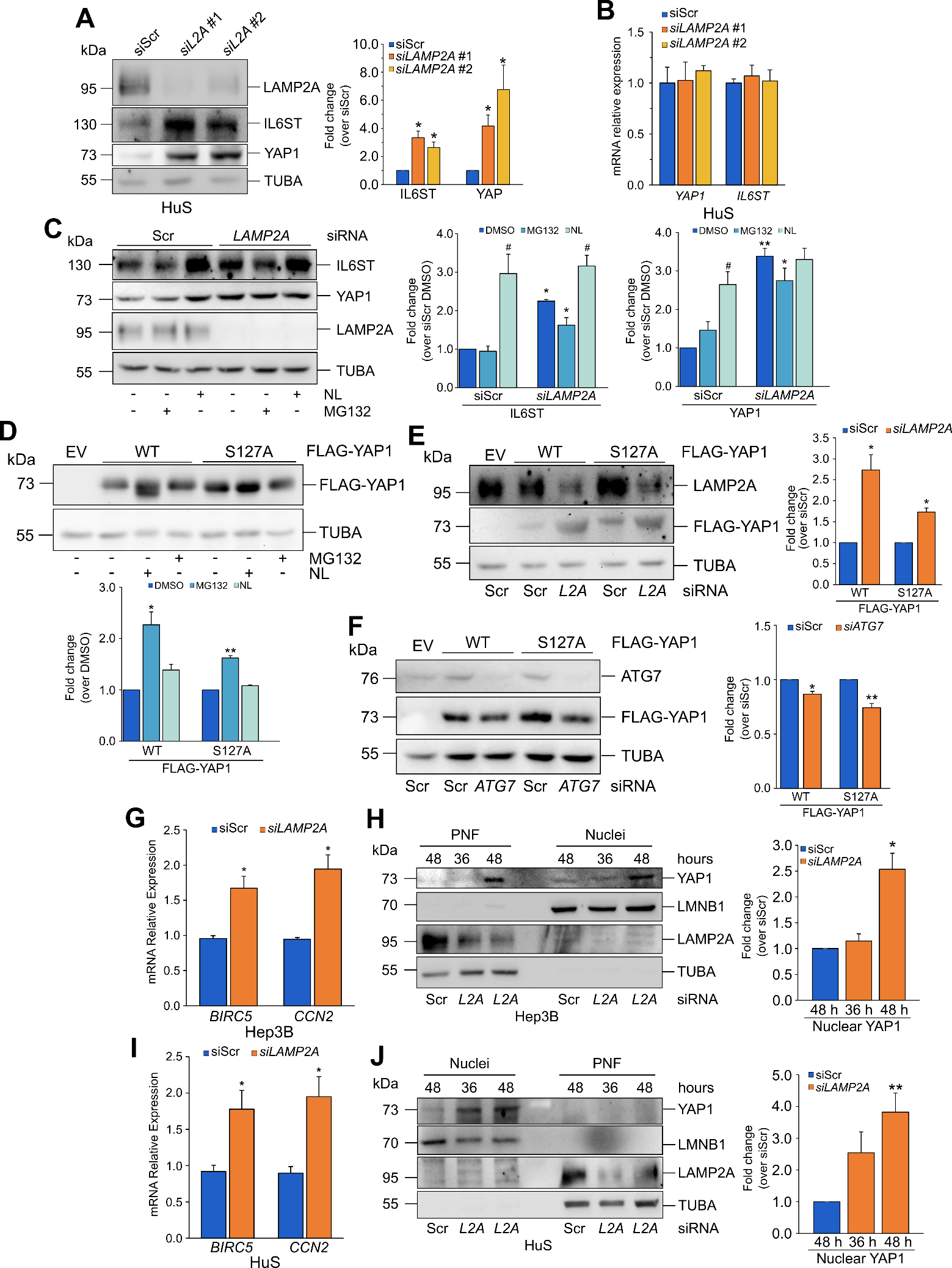
**

**Figure S2.** Modulation of CMA influences YAP1 and IL6ST protein levels and outputs. (**A**) Silencing of *LAMP2A* for 48 h causes the accumulation of YAP1 and IL6ST in HuS cells. The panel on the right shows the fold change of YAP1 and IL6ST over their expression in cells transfected with siScr, set as 1. (**B**) Silencing of *LAMP2A* for 48 h has no effect on the mRNA levels of *YAP1* and *IL6ST*. (**C**) Expression of YAP1 and IL6ST in siScr and *siLAMP2A* Hep3B cell treated for 6 h either with 5 μM MG132 or with lysosomal inhibitors (NL; 50 μM leupeptin and 20 mM NH_4_Cl). The panels on the right show the fold change of YAP1 and IL6ST over their expression in cells transfected with siScr and treated with DMSO, set as 1. (**D**) Expression of YAP1 WT and YAP1 S127A in Hep3B cell treated for 6 h either with 5 μM MG132 or with lysosomal inhibitors (NL; 50 μM Leupeptin and 20 mM NH_4_Cl). The bottom panel shows the fold change of YAP1 over the expression of the same construct in cells treated with DMSO, set as 1. (**E-F**) Silencing of *LAMP2A* (**E**) but not *ATG7* (**F**) increases the expression of both YAP1 WT and YAP1 S127A in Hep3B cells. The panel on the right shows the fold change of YAP1 over the expression of the same construct in cells transfected with siScr, set as 1. (**G,I**) Silencing of *LAMP2A* for 48 h increases mRNA levels of *CCN2* (YAP1 target gene) and *BIRC5* (common YAP1-IL6ST pathway target) in Hep3B (**G**) and HuS cells (**I**). mRNA levels were normalized using *ACTB* as housekeeping gene. (**H,J**) Silencing of *LAMP2A* increases nuclear localization of YAP1 in Hep3B (**H**) and HuS cells (**J**). LMNB1 and TUBA were used as controls of nuclear and post-nuclear (PNF) fraction, respectively. The panel on the right shows the fold change of YAP1 over the expression in cells transfected with siScr, set as 1. The intensity of the bands of interest was normalized to TUBA (panels **A, C** and **D**-**F**) or LMNB1 (panels **H** and **J**) prior to fold-change calculations. Data are expressed as the mean ± SEM of n=3 biological replicates. *p<0.05, **p<0.01 vs siScr. #p<0.05 vs DMSO. Related to Figure 2.

**
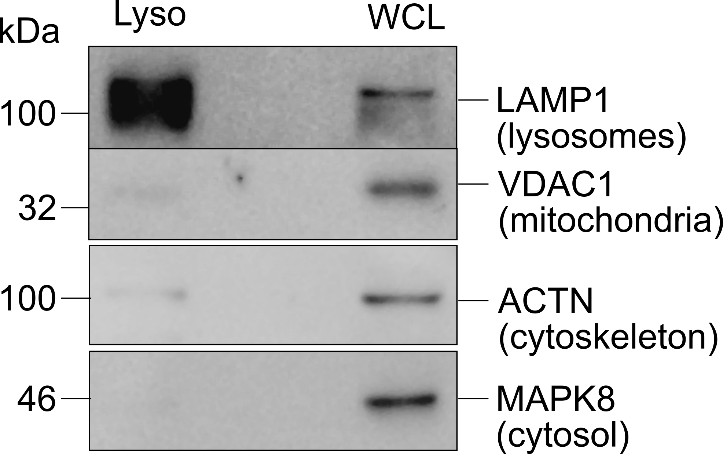
**

**Figure S3.** Purity of the lysosomal fraction obtained by density gradient centrifugation. Analysis of isolated lysosomes shows little to no contamination by other subcellular compartments. Lyso, lysosomes; WCL, whole-cell lysate. Related to Figure 3.


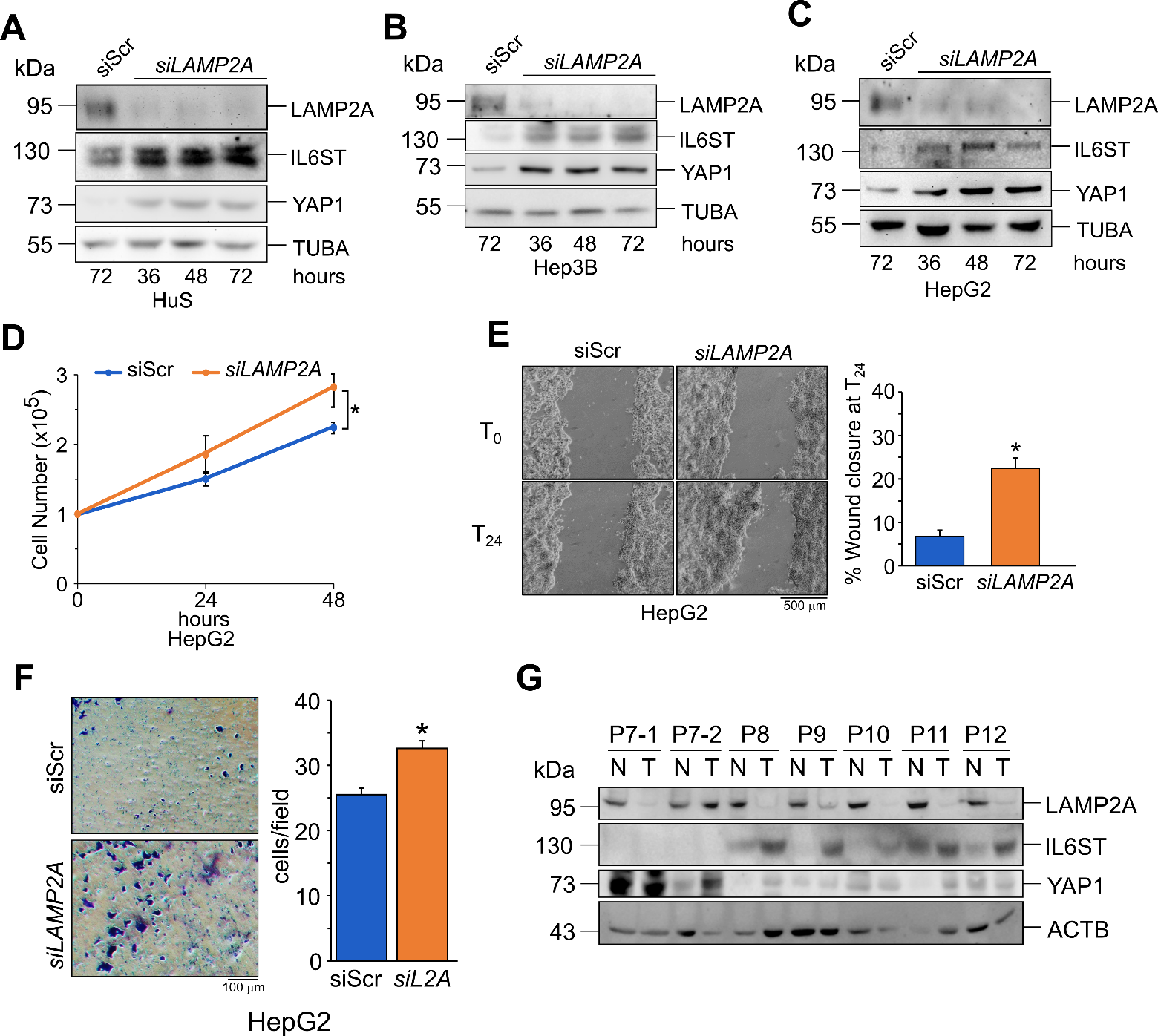


**Figure S4.** Inhibition of CMA promotes cell proliferation and migration in HepG2 cells. (**A,C**) Western blot analysis of LAMP2A, YAP1 and IL6ST in HuS (**A**), Hep3B (**B**) and HepG2 (**C**) cells transfected with *siLAMP2A* for the indicated times. (**D**) Inhibition of CMA increases proliferation of HepG2 cells, measured by live cell counting. (**E**,**F**) Inhibition of CMA increases migration of HepG2 cells, measured by the wound healing (**E**) and transwell (**F**) assays. Data are expressed as the ± SEM of n=3 biological replicates. *p<0.05 vs siScr. (**G**) LAMP2A, YAP1 and IL6ST protein expression in human HCC samples (T) compared to matched adjacent normal tissues (N). Related to Figure 4.


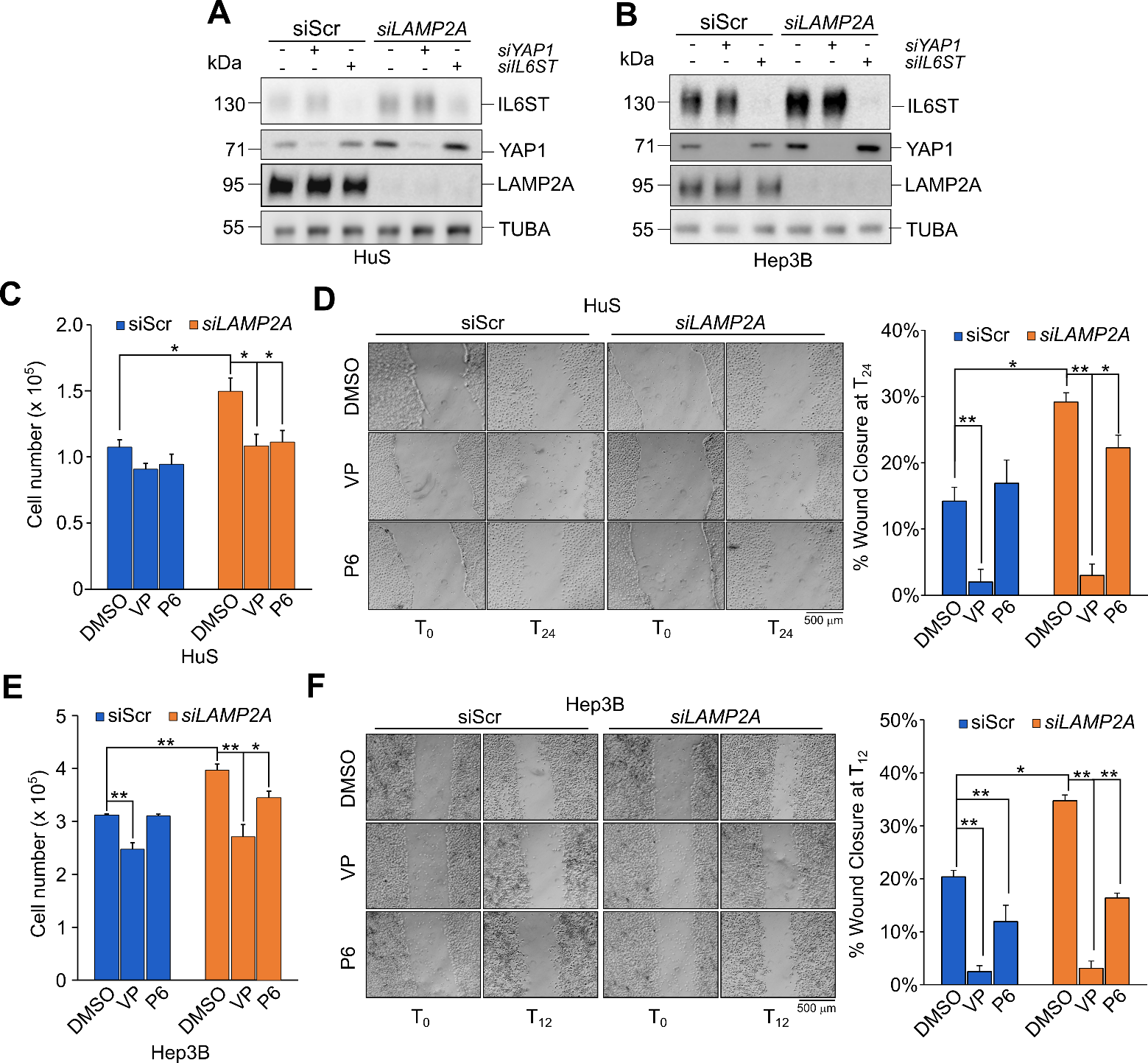


**Figure S5.** The pharmacological inhibition of YAP1 and IL6ST restrains the growth advantage and increased motility induced by CMA downregulation. (**A,B**) Western blot analysis of LAMP2A, YAP1 and IL6ST in HuS (**A**) and Hep3B (**B**) cells transfected with *siLAMP2A* and either *siYAP1* or *siIL6ST*. (**C,E**) Treatment either with 5 μM YAP1 inhibitor VP or with 1 μM JAK inhibitor P6 restrains the increased proliferation of *LAMP2A* knockdown HuS (**C**) and Hep3B (**E**) cells, measured by cell counting. (**D,F**) Treatment with either 5 μM YAP1 inhibitor VP or 1 μM JAK inhibitor P6 reduces the increased migration of *LAMP2A* knockdown HuS (**D**) and Hep3B (**F**) cells, as measured by an in vitro wound healing assay. Data are expressed as the ± SEM of n=3 biological replicates. *p<0.05; **p<0.01. Related to Figure 5.
